# Correlation-induced Suppression of Bilayer Splitting in High-*T*_*c*_ Cuprates: A Variational Cluster Approach

**DOI:** 10.1007/s10948-012-1537-x

**Published:** 2012-04-10

**Authors:** Anna M. Fulterer, Enrico Arrigoni

**Affiliations:** Institute of Theoretical and Computational Physics TU Graz, 8010 Graz, Austria

**Keywords:** High-temperature superconductivity, Electronic correlations

## Abstract

We carry out a theoretical study of the bilayer single-band Hubbard model in the undoped and in the superconducting phases by means of the variational cluster approach. In particular, we focus on the splitting between the “bonding” and “antibonding” bands induced by the interlayer hopping, as well as its interplay with strong correlation effects. We find that the splitting is considerably suppressed in both the normal and superconducting phases, in qualitative agreement with experiments on Bi_2_Sr_2_CaCu_2_O_8+*δ*_. In addition, in the superconducting phase, the shape of the splitting in *k* space is modified by correlations.

## Introduction

It is widely accepted that the fundamental physics of High-*T*_*c*_ superconductors (HTSC) takes place in the two-dimensional CuO_2_-layers. On the other hand, several classes of HTSC exist with a different number of CuO_2_-layers per unit cell, their transition temperature being strongly related to this number [1]. There have been several explanations for this phenomenon. Among them, one could mention interlayer interactions, charge imbalance, or quantum tunneling of Cooper pairs [2–4].

Experimental measurements, supported by theoretical investigations [5], show that the interlayer coupling and the third dimension more generally have a strong impact on angle-resolved photoemission spectroscopy (ARPES) results [6–8]. Depending on photon energy and polarization, different features are accentuated in the measured spectra [9, 10], while the “real” underlying quasiparticle spectrum remains hidden. In the last decade, the BiSrCuO compounds BSCO-2212 and BSCO-2201 have been studied thoroughly, and several conclusions have been drawn from the results: High resolution ARPES on BSCO-2212 with suppressed superstructure reveals the presence of two Fermi surface pieces: one hole-like, the other changing from electron to hole-like [10]. Heavily overdoped BSCO-2212 shows a difference in bilayer band splitting for the normal and superconducting case [11]. In the normal state, this is about 88 meV and gets renormalized to about 20 meV in the superconducting state. In the superconducting state, each one of the two split band develops its own peak-dip-hump structure (PDH). This is most probably due to the strong renormalization at about 60 meV produced by the interactions with spin fluctuations [11].

Bilayer splitting in the normal state only weakly depends on doping [12]. In optimally doped BSCO-2212 (bilayer), the quasiparticle in the (*π*,0) region should look similar to that of BSCO-2201 (monolayer) [9]; the enhanced linewidth in the bilayer material is attributed to correlation effects, more specifically (*π*,*π*) scattering due to antiferromagnetic fluctuations. In order to unravel the underlying mechanisms producing these effects, different theoretical methods have been applied. LDA calculation done for YBCO [13] show that the interlayer hopping comes from copper s electrons. Different models were used to describe the system of coupled 2D CuO planes, e.g., the bilayer Hubbard Model [14, 15], coupled two-leg spin ladders [16], tight binding extended Hubbard Model [17, 18], bilayer t-J model [19]. From these calculations, the following conclusions can be drawn. The PDH structure can be explained by a coupling of the electronic excitations to magnetic resonances or spin fluctuations [20, 21]. At low doping, the coupling between the layers should be antiferromagnetic [15], and there might be contributions to superconductivity by interlayer Cooper pairs, being formed by holes belonging to different layers. The reduction of the bilayer splitting with respect to the noninteracting tight binding model is attributed to the formation of spin bags in the layers [19], which increases the quasiparticle weight or/and antiferromagnetic interlayer order.

In this paper, we address these issues by an alternative approach in which correlations are evaluated exactly at a short-range level of a cluster, and thus is expected to capture the interplay between short-range antiferromagnetic coupling and quasiparticle excitations. Specifically, we use the Variational Cluster Approach (VCA) [22, 23] to solve the bilayer Hubbard model. VCA is an extension of Cluster Perturbation Theory (CPT) [24, 25]. Due to its variational nature, it allows for a treatment of symmetry breaking phases, in our case antiferromagnetism and/or superconductivity. The method has already been successfully been applied to a wide range of problems [23, 26–29] and is based on the Self-Energy Functional Theory (SFT) [30, 31].

We will illustrate the effects of bilayer splitting by displaying the spectral functions for the two bands. Finally, we will discuss the reduction of the splitting due to correlation in both the normal as well as in the superconducting state.

## Model and Method

A single CuO_2_ layer on the *x*–*y* plane is commonly described by the two-dimensional Hubbard Hamiltonian 
1 in standard notation. As usual, we include a next-nearest hopping in order to reproduce the band structure observed in ARPES experiments. As it is well known, for example, from LDA calculations, the interlayer hopping displays a characteristic *k* structure.^1^ Downfolding an 8-band Hamiltonian for the bilayer compound YBa_2_Cu_3_O_7_ (YBCO) [13] gives a $\vec{k}$-dependent interlayer hopping, originating mainly from copper s and oxygen d-orbitals in the form:

2 $$t_\perp(k) \approx\tilde{t} \frac{v^2}{(1-2ru)^2}$$

with 
3 and *u* another form factor, which we do not need to specify. *a* is the lattice constant on the layer, which for simplicity we take to be equal in the *x* and *y* direction. We take units in which *a*=1. However, since *r*≡*t*′/*t*=0.3≪1 , this term can be neglected and one obtains

4 $$t_\perp(k) \approx\frac{\tilde{t}}{4} (\cos k_y-\cos k_x)^2. $$

In our approach, we need the hopping term in real space. Fourier transformation yields three types of interlayer hopping terms, a vertical one *t*(Δ*x*=0,$\Delta y=0)=\tilde{t}/4$, a diagonal hopping (Δ*x*=±1, Δ*y*±1), and one along the *x* or *y* axis (Δ*x*=0,±2,Δ*y*=0,±2).

The method used for approximating the ground-state properties of the system is VCA. In a first step, the lattice is split up into identical clusters, which constitute the so-called reference system [32]. The model then solved exactly on each cluster, and the single particle Green’s function *G* ^*CL*^(*z*) of a cluster is calculated numerically, in our case by Lanczos exact diagonalization. The disconnected clusters are then coupled within strong-coupling perturbation theory at leading order in the hoppings, yielding an approximation for the Green’s function of the whole lattice in the form:

5 $$G^{CPT}(z)=\bigl(G^{CL}(z)^{-1}-T\bigr)^{-1},$$

where *T* is a matrix describing intercluster hoppings (see, e.g., [23, 29] for details).

A variational principle based on the self-energy functional approach has been formulated by Potthoff [32]. By introducing additional variational fields and “optimizing” the grand potential with respect to these fields, one can study broken-symmetry phases, such as magnetism or superconductivity [23, 26, 27]. Details of VCA can be found, e.g., in [26, 29]. In the present paper, we introduce the following variational fields, which within VCA are just used for the determination of the self-energy and then subtracted perturbatively [29]:

- staggered magnetic field
  6 $$H_M=h_M\sum_{i\sigma}(-1)^\sigma e^{i\vec{Q} \vec{r}} c_{i\sigma}^+c_{i\sigma},$$
  with *Q*=(*π*,*π*).
- superconducting field
  7 $$H_{SC}=h_{SC}\sum_{i,j}\frac{\eta_{i,j}}{2}(c_{i\uparrow}c_{j\downarrow }+c_{j\uparrow}c_{i\downarrow}),$$
  where *η* is the form factor which determines the symmetry of the superconducting order parameter, in our case d-wave.
- on-site energy
  8 $$H_{n}=\epsilon\sum_{i\sigma} n_{i\sigma}$$
  which is needed for thermodynamic consistency [29].

The nearest-neighbor hopping *t*=1 sets the energy scale, and we take typical values *U*=8 and *t*′=0.3*t* (see, e.g., [33]). The interlayer hopping is chosen to be $\tilde{t}\approx0.2$ close to the value estimated for BSCO-2212 in [18].

## Results

### Half Filling

**Fig. 1:**
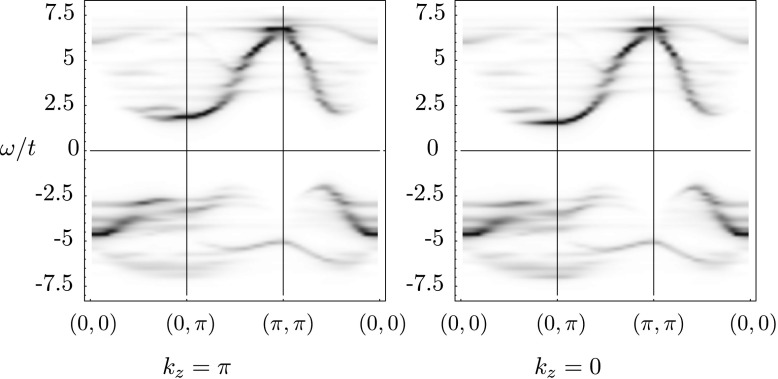
Spectral function *A*(*k*,*ω*) as a gray plot for the half filled bilayer Hubbard model. Results are shown for the bonding (*k*_*z*_=0) and antibonding (*k*_*z*_=*π*) band

**Fig. 2:**
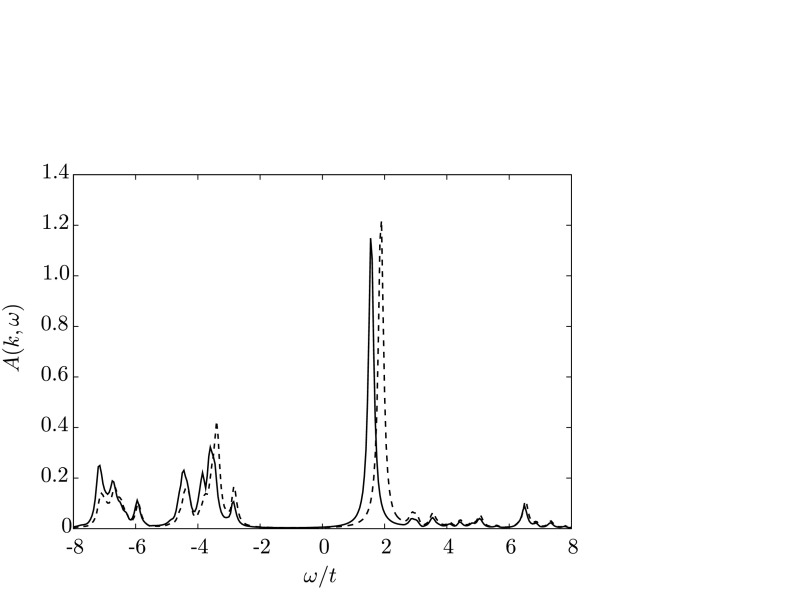
Spectral function at the (0,*π*) point (maximum bilayer splitting) for the bonding (*solid line*) and antibonding (*dashed*) bands

The spectral function *A*(*k*,*ω*) at half filling is plotted in Fig. 1 along the path [(0,0),(0,*π*),(*π*,*π*),(0,0)] in the two-dimensional Brillouin zone. The spectrum shows the asymmetric behavior of electron and hole filling induced by *t*′: electrons are expected to first enter the Brillouin zone around (*π*,0), while holes first enter at (*π*/2,*π*/2). The interlayer hopping introduces a splitting between the *k*_*z*_=0 and *k*_*z*_=*π* spectra, which for simplicity of language we will refer to as the bonding and antibonding bands [13]. Without correlations, we would expect the splitting of the bands to be given by $2*t_{\perp}(k)\frac{(\cos(k_{x} a)-\cos(k_{y} a))^{2}}{2}$. Looking at the Brillouin zone this means that along the diagonal *k*_*x*_=*k*_*y*_ the two Fermi points for the bonding and antibonding bands are exactly one over the other. When going away from this diagonal, the splitting grows until reaching a maximum near the (0,*π*) and (*π*,0) points. In Fig. 2, we plot the spectral function of the bonding and antibonding bands at (0,*π*), which clearly shows the interlayer splitting. The splitting is approximately *Δ*_*U*_=0.32*t*, which is reduced with respect to the value *Δ*_0_=0.4*t* in the noninteracting case.

### Optimal Doping

**Fig. 3:**
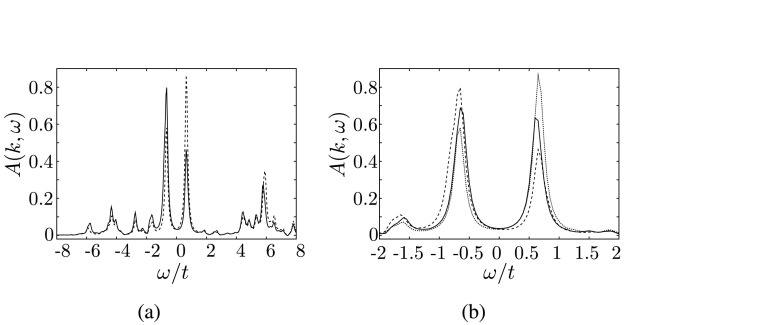
Spectral function at the (0,*π*) point for the optimally doped bilayer system (**a**) for the bonding (*solid line*) and antibonding (*dashed*) bands. (**b**) shows a comparison of the bilayer (*dashed*) with the monolayer (*solid line*) spectra

At optimal doping, no bilayer splitting could be resolved in ARPES measurements of BSCO-2212 [9]. In order to analyze this effect, the spectral functions for the bonding and antibonding bands at (*π*,0) in the superconducting case are displayed in Fig. 3(a) for optimal doping. Our calculations indeed suggest that the antibonding and bonding spectrum lie almost exactly over each other. Only differences in the peak strengths can be observed.

Moreover, it was found that the shape of the quasiparticle peak in the (*π*,0) region of the optimally doped monolayer (BSCO-2201) and bilayer material (BSCO-2212) are similar [9]. This is also very well reproduced in our data, as can be seen in Fig. 3(b).

### Overdoping

**Fig. 4:**
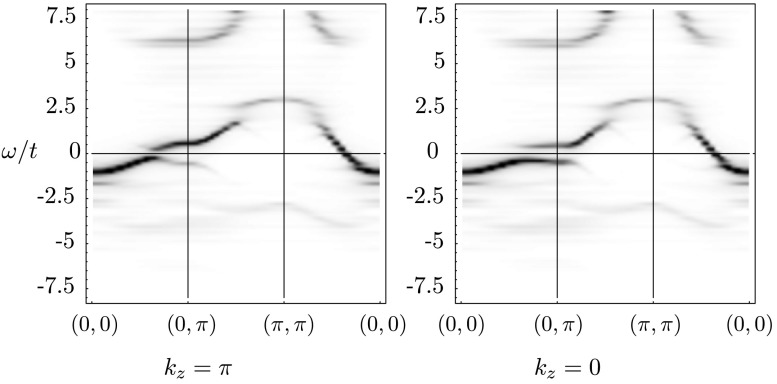
Spectral function *A*(*k*,*ω*) as a gray plot in the overdoped (*μ*=0.43) region in the superconducting phase

**Fig. 5:**
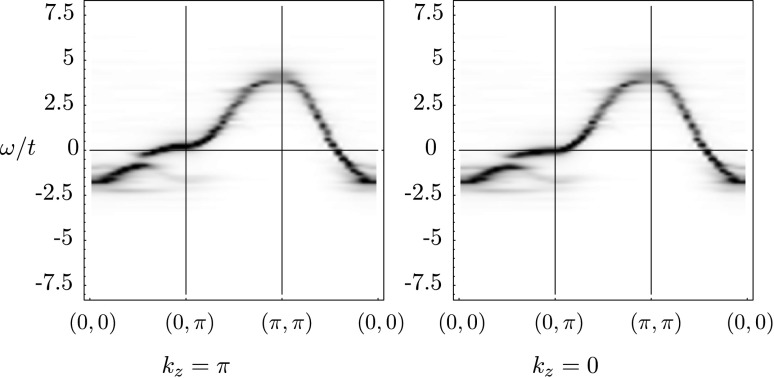
Spectral function *A*(*k*,*ω*) as a gray plot in the overdoped region in the normal phase

Bilayer splitting has been measured by ARPES in several works (see, e.g., [9–12]). In heavily overdoped samples, the splitting is suppressed much more in the superconducting case than in the normal state, contrary to the naive expectation that a global phase coherence below *T*_*c*_ will enhance the *c*-axis coupling, and thus cause larger splitting [11]. We checked these results by plotting the spectral function in the overdoped region^2^ of the bilayer Hubbard model both in the normal and superconducting state. These are displayed in Figs. 4 and 5.

**Fig. 6:**
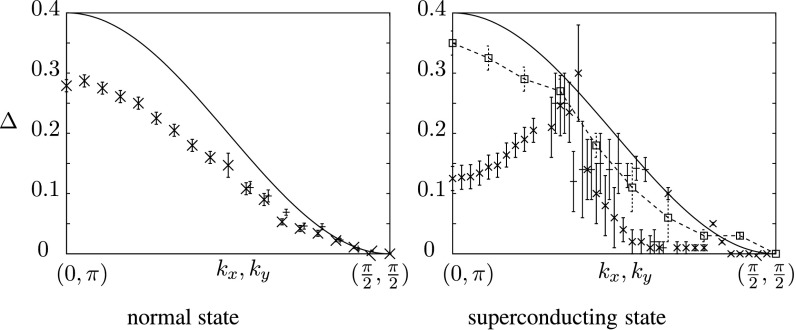
Energy bilayer splitting *Δ* along the *line* connecting (0,*π*) and (*π*/2,*π*/2) in the normal and superconducting state in the overdoped region (*crosses* and *lines* with *errorbars*). Results are compared to the splitting for *U*=0 (*solid line*). In the superconducting phase, we also display results obtained for *U*=0 by introducing “by hand” a superconducting symmetry breaking field (*dashed line*, *empty squares*). Away from the antinodal region, the excitations for both *k*_*z*_ bands develop a two peak structure similar to the peak–dip–hump that is seen in ARPES. Our results yield different results for the hump–hump and peak–peak splittings. For this reason, we display the second value using *thin errorbars*, shifted to the *right* for clarity. The *errorbars* represent the estimated error due to the uncertainty of the peak positions

In Fig. 6, we focus on details of the energy splitting and plot its *k*-dependence in the overdoped region. Our results suggest a reduction of the splitting at (0,*π*) by about 30 % in the normal and by about 70 % in the superconducting phase with respect to the tight-binding model. Moreover, in the superconducting phase, also the shape of the *k* dependence is modified. This larger suppression in the superconducting phase is in qualitative agreement with experiments [11]. In order to disentangle the effects of correlation from the ones due to the superconducting gap, we also display results obtained for *U*=0 by introducing “by hand” a superconducting symmetry breaking field equal to the one obtained variationally at *U*=8. As one can see from the figure, the superconducting gap only produces a small (about 10 %) reduction, which is uniform in *k*. The anomalous behavior of Fig. 6 is thus essentially due to correlations.

The values of the splitting for *U*=8 plotted in Fig. 6 are obtained in the following way: In the normal state, there is just one prominent dispersing peak for each *k*_*z*_ defining a bonding and antibonding band. The *k* dependent splitting is defined as the distance between the maxima of these peaks for *k*_*z*_=0,*π*. For the superconducting state, we determine the splitting for the quasiparticle states below the Fermi level. We have checked that it very close to the splitting of the mirror states above it. When going away from the antinodal point both in the normal state as well as in the superconducting state, each quasiparticle peak first broadens, which introduces an error in the determination of *Δ*, and then evolves into a two peak structure, which resembles the peak-dip-hump structure that is observed in ARPES [11]. Measuring the distance between the second pair of peaks gives a second set of data points, which is also displayed in Fig. 6.

## Conclusion

We have studied the bilayer Hubbard model by means of the variational cluster approach, a method appropriate to capture short range correlation in strongly interacting lattice systems. As expected, the interlayer hopping splits the spectrum into a bonding and an antibonding band. However, the corresponding bilayer splitting is strongly renormalized due to correlations. This is evident in the overdoped case in both the normal and superconducting phases. In qualitative agreement with ARPES measurements, the suppression effect is stronger in the superconducting phase. Surprisingly, for optimal doping, the bilayer splitting vanishes completely, as found in ARPES [9].

## Footnotes

1 For simplicity, we refer to *k* as the component of the crystal momentum parallel to the CuO_2_ layers (in units of the inverse lattice spacing 1/*a*). *k*_*z*_ is the perpendicular component and takes the values 0 for the bonding and *π* for the antibonding band, respectively.

2 We define it as the doping for which the SC order parameter is suppressed by about 10 %.
